# Experimental evolution of specialization by a microsporidian parasite

**DOI:** 10.1186/1471-2148-10-159

**Published:** 2010-05-28

**Authors:** Mathieu Legros, Jacob C Koella

**Affiliations:** 1 Laboratoire de Parasitologie Evolutive, CNRS UMR 7103, Université Pierre et Marie Curie Paris VI, 7 quai Saint Bernard, 75005 Paris, France; 2 North Carolina State University - Entomology Dpt, 840 Method Rd. Unit 1, Raleigh, NC 27607, USA; 3 Division of Biology, Imperial College London, Silwood Park Campus, Ascot SL5 7PY, UK

## Abstract

**Background:**

Evolutionary theory predicts that the pressure for parasites to specialize on one host or to become generalists on a wide range of hosts is driven by the diversity or temporal variability of the host's population and by genetic trade-offs in the adaptation to different hosts. We give experimental evidence for this idea by letting the parasite *Brachiola algerae *evolve on one of four genetically homogeneous lines of the mosquito *Aedes aegypti*, on a mixture of the four lines or on an alternating sequence of the four lines. The first regime was expected to lead to specialists, the other two to generalists. After 13 generations, we tested the evolved parasites on each of the four lines of the mosquito.

**Results:**

The specialized parasites were most infective on their own isofemale line and least infective on other isofemale lines, while the generalist parasites had intermediate infection success on all lines. The success of a specialist on its matched mosquito line was negatively correlated with its success on other lines, suggesting an evolutionary cost to specialization. This trade-off was corroborated by the observation that the generalists had higher average mean infectivity than the specialists over all isofemale lines.

**Conclusions:**

Overall, our experiment reveals the potential for specialization of a parasite to individual genotypes of its host and provides experimental evidence of the cost associated with the evolution of specialization, an important feature for understanding the coevolutionary dynamics between hosts and parasites.

## Background

Parasitic species' strategies of host exploitation fall into a very wide range, from specialists, which can infect and develop in a restricted variety of hosts, to generalists, which can use a wide range of hosts. Specialization on a given host is a selective process that increases the fitness of the parasite on this host. If the host population is homogeneous and remains constant through time, evolution is expected to favor parasites that are best adapted to this host, and therefore select for specialist strategies. On the other hand, the selective pressures operating in variable or heterogeneous host populations can mitigate this specialization process [1,2]. If evolution of the parasite is rapid with respect to the time scale of variation, temporal variation in a host population might lead to repeated evolution of parasites specialized on the different hosts, as has been observed for example in bacteriophages adapting to alternate hosts [3].

In a more rapidly changing or spatially heterogeneous host population, a parasite might find itself exposed to a variety of possible hosts. In this context, theory predicts that evolution favors parasitic strategies with the highest geometric mean fitness across different hosts [4]. Because this geometric mean increases as the variance of fitness over the hosts decreases [5], strategies associated with similar fitnesses across different hosts are positively selected.

The range of possible strategies is shaped by the assumption that specialization to one host is associated with a lower fitness on other hosts. This cost of adaptation is generally considered to be a necessary condition for the maintenance of diversity in heterogeneous environments [2,6]. In the case of parasitic species, an illustration of such a trade-off is the decreased ability of parasites to infect their original host after being maintained on a novel species for many generations [7].

While most studies of specialization of parasites deal with differences among host species (although there is a striking lack of experimental evidence [8-10]), the ideas should also be valid for genetic differences within a single host species. Trade-offs in genotype-specific specialization have been demonstrated in microbial [11] as well as eukaryotic [12-14] systems. We provide direct experimental confirmation of these ideas and quantification of this trade-off with the microsporidian *Brachiola algerae *(formerly *Nosema algerae *), a common insect parasite [15], and one of its hosts, the yellow fever mosquito *Aedes aegypti*. *B. algerae *spores infect host larvae when they are ingested, and the parasite then proliferates within host tissues as the larva grows. Mortality in *A. aegypti *due to *B. algerae *microsporidiosis is usually quite low, while the parasite's virulence is rather reflected by a delay of the host's pupation. We let parasites evolve in three types of regimes differing in the combinations of the mosquito's genotypes (consisting of four isofemale lines). First, in four *single line *regimes, the parasites were maintained on only one of the four isofemale lines. Second, in a *mixture *regime the parasites were maintained on an equitable mixture of the four lines, providing a regime where host population is heterogeneous. Third, in an *alternating *regime, the parasites were maintained on a single isofemale line in any given generation, but the line was alternated in a regular sequence among generations. After thirteen generations, we tested the parasites of each of the evolved parasite lines on each of isofemale host line by measuring the parasites' infectivity (the proportion of mosquitoes that were infected) and the number of spores produced in infected individuals as two components of the parasite's transmission success. The first (*single line*) regime is expected to select for specialist strategies. If specialization is costly, the performance of specialized parasites on other host lines is expected to be significantly lower, while parasites evolved in the second and third regimes should exhibit intermediate success across different host lines.

## Results

**Figure 1:**
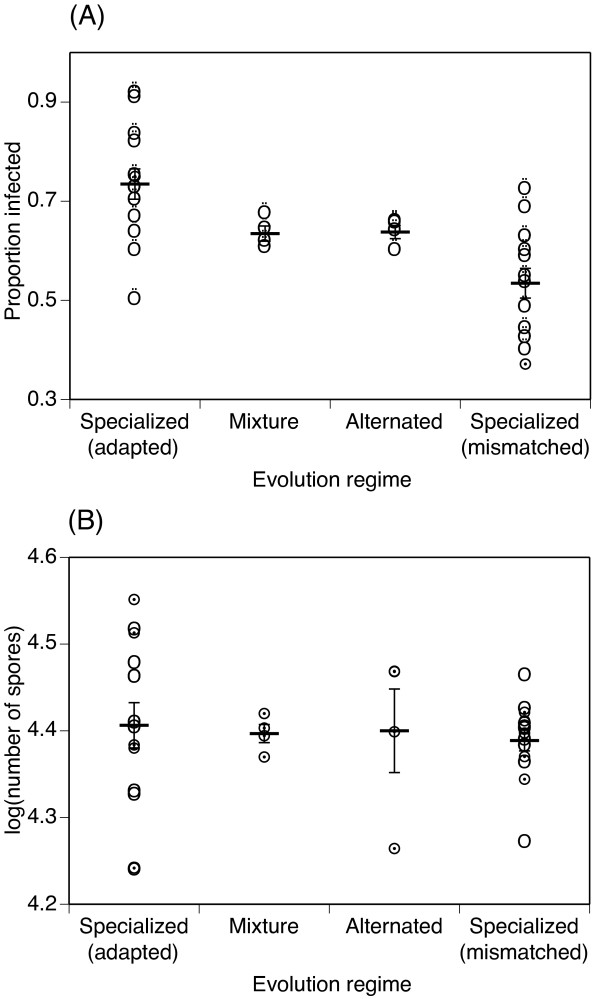
**Test of evolved parasites**. **(A) **Infectiousness of the evolved parasites and **(B) **number of spores found in infected mosquitoes when the parasites were tested on the four isofemale lines of the mosquito host. 'Specialized' refers to parasites that had evolved on one isofemale line; 'adapted' refers to parasites that were tested on the same line while 'mismatched' refers to parasites that were tested on a different line. 'Mixture' refers to parasites that had evolved on a mixture of the 4 isofemale lines. 'Alternated' refers to parasites that had evolved on an alternating sequence of the 4 isofemale lines. Each point gives the parasites' infectivity (A) or the mean log-transformed number of spores in infected mosquitoes (B), and the horizontal line gives the means of the pooled replicates. In (A) the vertical lines give the 95% confidence interval of the proportion, and in (B) they give the standard error of the mean.

After 13 generations of evolution, the parasites had developed clear differences in infectivity (Fig. 1a, Table 1a, P < 0.001). Thus, *adapted *parasites (i.e. parasites that had evolved on a single line and were tested on the same line) infected 73.7% (± 2.5% standard error among replicates) of the larvae, parasites that had evolved in a *mixture *of isofemale lines infected 63.4% (3.6% S.E.), parasites that had evolved on an *alternating *sequence of isofemale lines infected 63.6% (3.8% S.E.), and *mismatched *parasites (evolved on a single line and tested on other lines) infected 53.5% (0.2% S.E.). The two regimes that were expected to lead to generalists (*mixture *and *alternating*) did not differ significantly in their infectivity, but both differed from *adapted *and *mismatched *parasites. In contrast, when we considered only the mosquitoes that were infected, we detected no significant differences in the log-transformed number of spores among the four types of parasites (median number of spores = 25 × 10^3^; Fig. 1b, Table 1b, P = 0.288).

**Table 1:** Analysis of (A) infectivity and (B) (log-transformed) number of spores in infected individuals for the different test combinations.

		(A) Infectivity		(B) Number of spores in infected individuals		
	**df**	**χ^2^**	**P**	**SS** **(× 10^3^)**	**F**	**P**

Test combination	3	28.60	< 0.001	194	1.26	0.288
Evolution regime [Test combination]	6	11.90	0.064	326	1.05	0.390
Replicate [Test, Evolution regime]	25	22.35	0.615	1262	0.98	0.497
Error	531			57438		

The analysis compares the four types of tests: specialized parasites on their own host, specialized parasites on mismatched hosts, parasites evolved on mixtures of hosts and parasites evolved on alternating hosts. 'Evolution regime' refers to the isofemale line (or combination of lines) the parasites evolved on.

To test more formally whether the parasites had indeed specialized on their hosts, i.e. whether they adapted to the hosts they had evolved on, we compared the *adapted *parasites with the *mismatched *ones. The analysis was done in two steps (see Methods). First, we performed a logistic analysis (for infectivity) or ANOVA (for number of spores) that included the interaction between selection line and test line. In a second step, we split the interaction term into two components, the first describing the difference between adapted and mismatched combinations and the second accounting for the rest of the interaction (Table 2). The analysis showed that the difference between *adapted *and *mismatched *parasites explained a significant component of the variation of the parasites' infectivity (P < 0.001), confirming that the parasites had indeed evolved to be more infectious on the isofemale line they had evolved on. However, as in the first analysis we did not observe any specialization concerning the number of spores within infected hosts.

**Table 2:** Type-1 statistical analysis of (A) infectivity and (B) (log-transformed) number of spores in infected individuals.

		(A) Infectivity		(B) Number of spores in infected individuals		
	**df**	**χ^2^**	**P**	**SS**	**F**	**P**

Test line	3	11.22	0.01	1.148	1.39	0.246
Isofemale line	3	4.53	0.209	0.244	0.30	0.828
Replicate [Isofemale line]	10	5.62	0.846	3.172	1.15	0.323
Test line* Isofemale line	9	24.33	0.004	1.994	0.80	0.612
***Adapted***	***1***	***20.16***	***< 0.001***			
Test line * Isofemale [Adapted]	*8*	*4.24*	*0.835*			
Testline * Replicate [Isofemale line]	30	29.00	0.517	5.521	0.67	0.909
Adapted*Replicate [Isofemale *line]*	*10*	*10.51*	*0.397*			
Error	311			85.632		

The analysis compares only specialized parasites tested on their own host and specialized parasites tested on mismatched hosts. 'Isofemale line' refers the isofemale line the parasites evolved on; 'Test line' is the isofemale line the evolved parasites were tested on. In (A), the factors written in italics are the components of the previous interaction (see Methods.) Note that the second component of the interaction Testline * Replicate is pooled into the error term. The component 'Adapted' (in bold) gives the importance of specialization onto a given host line. In (B) we did not decompose the interaction terms, as they were far from statistically significant.

**Figure 2:**
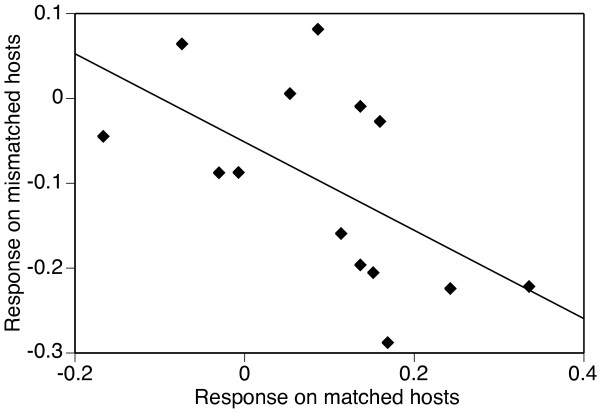
**Visualization of the cost of specialization**. Each point represents the value of the response index of an evolved parasite on its matched host (i.e. on the isofemale line it had evolved on) against the mean value on its three mismatched hosts. The response index is given as the relative difference between the infectivity of specialized parasites and the infectivity of generalist parasites tested on the same isofemale line. Specialization is costly if the parasites performing best on matched hosts perform worst on mismatched hosts.

The cost of specialization is illustrated in Fig. 2, where we plot, for each parasite, its mean index values on the three mismatched hosts (to avoid pseudo-replication) against its value on a matched host. Our results show a negative correlation between the two types of index values (df = 13; r^2 ^= 0.34; p = 0.029), confirming the cost of specialization in this system. (Note that we did not calculate an equivalent index or estimate the cost of specialization for the number of spores in infected mosquitoes, as the first analyses showed no evidence of an evolutionary response of this parameter.)

## Discussion

Our results confirm the idea that homogeneous and constant host populations let parasites evolve to be specialists on certain genotypes of the host, while diverse or temporally variable populations lead to the evolution of generalist strategies. It is striking that this specialization is reflected in the probability that the parasites infect their host, but not in the number of spores (which is expected to be linked to the potential for further transmission) once the host is infected. This might correspond to the lack of a known effective immune response against microsporidian parasites in insects (although several aspects of the immune system of mosquitoes are stimulated or suppressed by microsporidians [16]). Alternatively, a rapid innate response could be responsible for the difference in results for infectivity and for the number of spores in successful infections.

In addition, our results confirm that specialization to a given host is associated with being less well adapted to alternative hosts. This negative correlation is expected under the hypothesis of an evolutionary cost of adaptation, i.e. that increased success on one type of hosts is associated with decreased success on other (unrelated) hosts. Previous studies (e.g. [11-13]) have observed such a cost, and indeed it seems likely that in the long term there must be a cost of adaptation [9], although this is not always observed [17,18]. It should be noted that our experimental design does not allow us to demonstrate what evolutionary processes led to the observed differences in infectivity. In particular, our interpretations are limited by the impossibility to preserve the ancestral parasite strain throughout the time of the experiment. Because *B. algerae *spores cannot be viably stored or frozen for a long period of time, they have to be maintained on their hosts, which renders the true ancestral state unavailable. We are also limited in our knowledge of the existing diversity within the microsporidian population, which hinders our understanding of the evolutionary processes at work in this study. Therefore, we interpret the observed trade-off as a cost only in the general sense that infectivity on non-passaged lines is lower than that on passaged lines, but cannot attribute these differences specifically to an increased infectivity of specialists on the line in which they were passaged, a decreased infectivity on the other lines, or a combination of both.

These conclusions rely on the critical assumption that parasites that had evolved on a mixture of hosts behave as one generalist strain, as opposed to a mix of specialized strains. Without a direct assessment of the genotypic diversity within that strain, it is impossible to disprove this alternative hypothesis. We believe however that we are indeed in presence of a generalist strain, based on two main observations. First, the results remain qualitatively similar when we use the temporally variable *alternating *regime as a source of generalists, a regime intuitively less likely to maintain a mix of specialists from generation to generation. Second, if this strain was in fact a mix of specialist strains, it should be able to infect each isofemale line as effectively as the best adapted specialist. Therefore, as long as infectivity is not limited by the concentration of spores, such a mix of specialists would not explain the observed lower infectivity. We chose our spore concentration (3000 spores.cm^-2^) to be fairly high to avoid as much as possible these potential dose effects, so that our preliminary tests as well as other studies [19] confirm that lower concentrations lead to similar levels of infectivity.

One important caveat of this interpretation comes from the limited number of generations that we used in our selection treatments. An alternative explanation for the reduced infectivity of parasites evolved on a mixture of hosts is that they have had less time to adapt to each of their hosts [20]. Under this hypothesis, even without any cost to adaptation, it is expected that parasites selected on a mixture of hosts would initially exhibit a lower success on a single host line because of the lower time spent within this host, but ultimately reach similar infectivity if selection is carried out for a long enough time. Therefore, the observed pattern could be due to the difference in true selection time rather than to a cost of adaptation if the duration of our selection phase (13 generations) falls in the range during which such transient differences would be observed. Our choice of 13 generations was limited by practical considerations, and the relatively long generation time of our mosquito host for experimental evolution studies. To discriminate between these hypotheses, similar experiments could be carried out with an extended selection period. If there is a cost of adaptation, differences in infectivity will hold no matter how long selection acts, while under the alternative hypothesis of differences in time on each host, longer selection would eventually lead to similar infectivities.

If the observed differences are indeed due to a cost of specialization, the underlying genetic mechanism could be antagonistic pleiotropy, where the alleles responsible for the adaptation to one environment are detrimental in other environments [21], or accumulation of mutations that have no or an only slightly deleterious effect in the evolved environment, but are strongly deleterious and thus become an important load in other environments [22]. Both mechanisms have received empirical support from other studies of ecological specialization [23,24]. Although our experiment does not enable us to discriminate between the two possibilities, the accumulation of deleterious mutations seems less probable, as the number of mutations is limited by a small number of generations and by a bottleneck of the size of the parasite's population when hosts ingest the parasite's spores. Therefore, it is more likely that the trade-off revealed in our experiment is based on antagonistic pleiotropy of the alleles involved in the adaptation of *B. algerae *to its host.

Because of the cost of specialization, generalist strategies should be expected to have higher average success in population with diverse hosts. Indeed, if we pool the data from the four isofemale lines, the parasites that had evolved in the two generalist regimes (*mixture *and *alternating*) tended to have higher infectivity on average (63.5% ± 2.6% standard error) than the specialist parasites (58.5% ± 1.9% s.e.), although this difference was not quite statistically significant (logistic analysis: χ^2 ^= 3.00, df = 1, p = 0.083). However, the long-term fitness of our parasites in heterogeneous environments should be assessed by the geometric mean fitness (GMF) rather than the above average infectivities. Although this GMF cannot be calculated here without measuring fitness across several generations, one might observe that the specialist strategies are associated with higher variance of infectivity across the genotypes of the host (Fig. 1). Given that GMF decreases with the variance of the fitness across generations [5], we can speculate that the above difference in average infectivity is an underestimation of the actual difference in GMF between specialists and generalists. Thus, specialization on a specific genotype does not pay in a heterogeneous or varying environment, enabling generalist strategies to dominate populations.

## Conclusions

Overall, our experiment provided experimental support for the two main ideas about the evolution of specialization by giving two clear results. First, the microsporidian parasite had a high potential for specialization on individual genotypes of its host. Indeed, parasites that had evolved for thirteen generations on a specific isofemale line of their host were about 10% more likely to infect this isofemale line than if they had evolved on a mixture of isofemale lines. Second, the adaptation to a given host genotype was costly in that it led to a lower infectivity on other isofemale lines. Because of this cost of specialization, generalist strategies were, on average, more successful over the four mosquito lines used in the experiment. This cost, preventing the evolution of a generalist that would be superior on all genotypes, could explain the high level of specificity generally observed in invertebrate host-parasite interactions [18,25,26].

## Methods

### Experimental evolution

Each of the evolution regimes mentioned above was replicated four times. In the *alternating *regime, each replicate was initiated with a different isofemale line. Note that two replicates of the specialization regime on one of the isofemale lines were lost after three generations and were reinitialized. As the parasites on these isofemale lines had been given less opportunity to adapt, the results are presented without these two delayed replicates. However, including them in the analysis did not change the results qualitatively.

The isofemale lines of the mosquito were derived from a laboratory colony of *A. aegypti *maintained by J.J. Becnel (USDA, Gainesville, USA), had been maintained for more than 50 generations, and have previously been shown to differ in several life history traits and in their immune response (unpublished Masters theses). A stock of the parasite *B. algerae *was obtained from M.H.H. Hansen (University of Aarhus, Denmark). Throughout the passaging experiment, we flooded eggs under reduced pressure for 30 min to synchronize the hatching of the mosquitoes, and reared 20 larvae in Petri dishes (5.5 cm diameter). The larvae were fed on TetraMin™ (day 1: 0.04 mg/larva, day 2: 0.08 mg, day 3: 0.16 mg , day 4: 0.32 mg, from day 5 onwards: 0.16 mg, all amounts per larva). On day 1 they were exposed to 3000 spores/cm^2 ^of the microsporidia added to the water. To obtain the parasites used to infect the next generation of mosquitoes, we collected the dead larvae and the pupae before they emerged as adults, crushed them in demineralized water to release the spores, and used this solution to infect the next generation of hosts.

### Test of evolution response

Immediately after the thirteenth generation of passaging, we assayed the parasite's infectivity (the proportion of mosquitoes that were infected) on various host lines and the number of spores produced in infected individuals, two important components of the parasite's transmission success. Each of the 22 evolved parasite lines was assayed on 12 individuals of each of the four isofemale host lines. For each test, we reared 12 larvae individually in 12-well plates and exposed them to 3000 spores/cm^2 ^of the evolved microsporidia. We collected the dead larvae and pupae and assayed the success of the parasite by counting the infected hosts and estimating the number of spores within them in a haemocytometer.

### Statistical analyses

In a first crude analysis (Table 1) of the evolution responses, we compared four types of tests: (1) parasites that had evolved on a *mixture *of host lines and were tested on any single one of these host lines, (2) parasites that had evolved on an *alternated *sequence of host lines and were tested on any single one of these host lines, (3) *adapted *parasites, which had evolved on a single host line and were tested on that same line, and (4) *mismatched *parasites, which had evolved on a single host line and were tested on a different one. We analyzed infectivity (proportion of larvae that were infected) with a logistic analysis, and number of spores in infected individuals with an analysis of variance. In both tests, evolution regime (*i.e*. the isofemale line(s) on which parasites had evolved) was nested within the type of test, and the replicate within each evolution regime. In the analysis of variance, the number of spores was log-transformed so that the requirements of the test were met.

As a second analysis (Table 2), which gave a better indication of specialization, we conducted a standard type-1 two-way ANOVA, considering only the specialized parasites (tests (3) and (4) above), and using as main factors the single isofemale line the parasites had evolved on, and the line they were then tested on (extended with replications within the line the parasites had evolved on). The interaction between the line used for the evolutionary process and the test line describes the variance due to the differences among the 14 parasite line x mosquito line combinations (16 when the two delayed lines are included). To evaluate the specialization, we divided these combinations into 4 cases where the parasite was *adapted*, and 10 (resp. 12) where the parasite was *mismatched*. Thus, with a dummy variable that coded for *adapted *or *mismatched *(factor 'Adapted' in Table 2), the interaction term could be split into the component responsible for the difference between *adapted *and *mismatched *combinations, and into the remainder of the interaction. The interaction describing the replicates could be split similarly, with the remainder added to the error term of the analysis.

Finally, to estimate the cost of specialization, we defined an index of the evolutionary response of specialized parasites as the relative difference between infectivity of parasites that had evolved in a *single *regime and were tested on one of the host's isofemale lines, and the infectivity of generalists (that had evolved on a *mixture *of hosts) on the same isofemale line. If we used parasites that had evolved on an *alternating *regime as generalists for this index, we obtained similar results. The cost of specialization can then be estimated by the correlation between each parasite's mean index values on the three mismatched hosts (to avoid pseudo-replication) and its value on a matched host. If specialization is costly, the best adapted parasites, exhibiting the highest index values on matched hosts, will be least infective (and thus have the lowest index values) on mismatched hosts.

## Competing interests

The authors declare that they have no competing interests.

## Authors' contributions

ML and JCK designed the experiments, analyzed the data and wrote the manuscript. ML performed the experiments. All authors read and approved the final manuscript.
